# Measuring Effects of Two-Handed Side and Anterior Load Carriage on Thoracic-Pelvic Coordination Using Wearable Gyroscopes

**DOI:** 10.3390/s20185206

**Published:** 2020-09-12

**Authors:** Sol Lim, Clive D’Souza

**Affiliations:** 1 Department of Systems and Industrial Engineering, The University of Arizona, Tucson, AZ 85721, USA; 2 Department of Industrial and Operations Engineering, University of Michigan, Ann Arbor, MI 48109, USA; crdsouza@umich.edu

**Keywords:** load carriage, gyroscope, gait kinematics, gait detection, thoracic-pelvic coordination, wearable sensor

## Abstract

Manual carrying of heavy weight poses a major risk for work-related low back injury. Body-worn inertial sensors present opportunities to study the effects of ambulatory work tasks such as load carriage in more realistic conditions. An immediate effect of load carriage is reflected in altered gait kinematics. To determine the effects of load carriage mode and magnitude on gait parameters using body-worn angular rate gyroscopes, two laboratory experiments (*n* = 9 and *n* = 10, respectively) were conducted. Participants performed walk trials at self-selected speeds while carrying hand loads in two modes (two-handed side vs. anterior) at four load levels (empty-handed, 4.5 kg, 9.1 kg, and 13.6 kg). Six measures of postural sway and three measures of thoracic-pelvic coordination were calculated from data recorded by four body-worn gyroscopes for 1517 gait cycles. Results demonstrated that, after adjusting for relative walking speed, thoracic-pelvic sway, and movement coordination particularly in the coronal and transverse planes, characterized by gyroscope-based kinematic gait parameters, are systematically altered by the mode of load carriage and load magnitude. Similar trends were obtained for an anthropometrically homogenous (Expt-1) and diverse (Expt-2) sample after adjusting for individual differences in relative walking speed. Measures of thoracic-pelvic coordination and sway showed trends of significant practical relevance and may provide sufficient information to typify alterations in gait across two-handed side vs. anterior load carriage of different load magnitudes. This study contributes to understanding the effects of manual load carriage on thoracic-pelvic movement and the potential application of body-worn gyroscopes to measuring these gait adaptations in naturalistic work settings.

## 1. Introduction

Load carriage is common in daily activities. People often choose to carry loads in their hands in an anterior or lateral location for convenience over short distances or for intermittent periods. Manual load carriage is also prevalent and inevitable in many occupations such as military [1], firefighting [2], and construction work [3], and for Manual Material Handling (MMH) work in manufacturing [4], warehousing [5], packaging, and distribution [6,7]. In a comprehensive study of MMH conducted at 2442 industrial locations, load carriage represented 15.7% of all MMH tasks recorded and was superseded only by lifting-lowering [8]. Routine load carriage is associated with an increased risk of work-related Musculoskeletal Disorders (MSDs) in the back [9,10] and upper and lower limbs [11,12]. A study investigating the relationship between load carriage and low back MSD prevalence among steel-workers found that frequent load carriage of objects weighing 5 to 15 kgs was highly associated with low back pain and injury with an odds ratio of 7.2 (95% CI: 1.60–32.4) [13]. Prior studies associate the increase of hand loads to increased torso and hip moments [14,15,16], increased back and abdominal muscle activity for improved spinal stability [17,18,19], and increased compressive and shear loads on the lumbar spine [3,20,21,22].

Beyond temporal aspects of duration and repetition, the risk from load carriage to the musculoskeletal system also depends on the load magnitude and the mode or manner by which the load is carried [22,23]. The latter alters the position of the load relative to the spine thus affecting compressive and shear stresses on the spine. Prior studies have quantified the increased risk of spinal injury from load carriage by using analytical models to estimate spinal loads. McGill et al. [20] estimated spinal compressive loads to be significantly higher when carrying weights in one hand compared to both hands. Rose et al. [22] investigated compressive and shear loads in the lumbar spine in various modes of load carriage including one-handed side carry, shoulder carry, two-handed anterior carry, and posterior carry. They concluded that, for the same load level, two-handed anterior carry resulted in the largest increase in anterior-posterior shear loading and thereby posed the greatest relative risk of low back injury [22]. Rohlmann et al. [24] estimated that lumbar compression force with respect to standing was nearly twice as high for carrying a weight in front of the body compared to carrying it laterally.

Human gait encapsulates movement with the arms, head, legs, and torso. The movements of each segment may seem variable and arbitrary, but, in fact, are repeatable and predictable. This is particularly true of kinematic adjustments for maintaining postural stability in response to external hand loads while walking. Effects of posterior loads such as from carrying a backpack or rucksack on gait patterns [25,26,27,28] has received the most attention due to its relevance to certain vulnerable populations, e.g., military soldiers [29,30], firefighters [2], and school children [31,32]. Increasing backpack load was associated with a decrease in swing duration [33], single support duration [28], and stride length [25], and an increase in double support duration [28] and stride frequency [25]. Increasing posterior loads also produced changes in postural kinematics such as increased mean torso inclination [27,28,30], decreased torso angular acceleration [14], and decreased transverse torso and pelvis rotation [25].

Biomechanical loading from load carriage also affects the way people move their torso and pelvis when walking. Relative phase angle, described by the phase difference or relative phase between two oscillating segments, provides a measure of coordination between multiple body segments or joints during complex and repetitive multi-joint movements [34]. In conventional gait analysis, relative phase angles are used as a measure of rotational thoracic-pelvic coordination [16,25,35,36,37]. Graham et al. [38] used variability in thoracic-pelvic relative phase angles as a measure of postural stability to compare posterior vs. anterior load carriage. Phase angle relationships between torso and pelvis may provide sufficiently sensitive measurements to characterize changes in intersegment coordination induced by alterations in load moment associated with task variables, specifically object mass and carrying mode.

A limitation of prior studies using intersegment relative phase angles is that they relied on optical motion tracking with participants walking at discrete and precise speeds on a treadmill. These studies do not directly extend to naturalistic gait speeds adopted by workers in applied work settings. Wearable inertial sensors, which include accelerometers, inclinometers, gyroscopes, and inertial measurement units (IMUs), have gained considerable attention in biomechanics research as an inexpensive and less obtrusive form of bioinstrumentation [39]. The portability and low power consumption of inertial sensors make them suitable for monitoring ambulation under naturalistic conditions, potentially outdoors and over long durations [40,41]. Multiple studies have demonstrated the validity and reliability of commercial, body-worn inertial sensors and related data processing algorithms using data from angular rate gyroscopes and/or linear accelerometers to analyze able-bodied and pathological gait [42,43,44,45,46]. In comparison, the application of body-worn inertial sensors to analyze ambulatory occupational tasks such as MMH and load carriage is still emerging [47].

The objective of this study was to quantify and compare the effects of a two-handed side and anterior load carriage performed at self-selected walking speeds on the amplitude and coordination of thoracic and pelvic rotations obtained from body-worn gyroscopes. The magnitude and mode of load carriage were hypothesized to influence torso and pelvic sway and thoracic-pelvic coordination during load carriage relative to unloaded gait after accounting for individual walking speed. To demonstrate generalizability of the findings, two separate experiments with similar methods were conducted. The experiments differed in the homogeneity in the sample composition and the make/brand of commercial wearable sensor used, and are presented separately. Consistent results from the two experiments were considered to indicate converging evidence, while inconsistent results could reflect effects specific to a particular experiment design. Previously validated algorithms from the biomechanics literature were adapted for extracting gait parameters from body-worn gyroscopes and are summarized in the appendices.

## 2. Experiment 1

### 2.1. Materials and Methods

#### 2.1.1. Participants

The study sample in Experiment-1 comprised nine healthy male individuals aged between 18 to 35 years old. Prior to participation, participants provided written informed consent and were screened for pre-existing back injuries or chronic pain with a body discomfort questionnaire adapted from the body mapping exercise developed by NIOSH [48]. Gender and health restrictions were applied in order to minimize potential inter-subject variability in gait patterns from these sources in the interest of obtaining a homogeneous sample. Participants’ stature and mass were measured to calculate Body Mass Index (BMI; kg/m^2^). The study was approved by the University of Michigan’s institutional review board.

#### 2.1.2. Experiment Procedure

**Figure 1:**
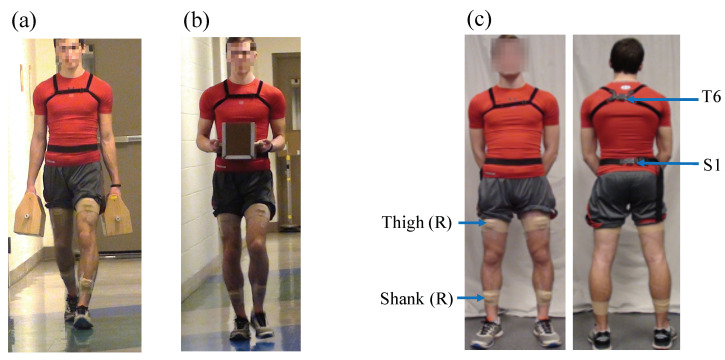
Images showing the two carrying modes evaluated in this study: (**a**) two-handed side carry, (**b**) two-handed anterior carry, along with the location of (**c**) four inertial sensors attached on the body at T6 (Posterior), S1 (Posterior), thigh (Right), and shank (Right).

A laboratory experiment was conducted that required participants to carry a weighted box on a straight, marked path (26.2 m length × 1.6 m width) with a levelled-floor for a distance of 24 m in two carrying modes, namely, a two-handed side carry and two-handed anterior carry (Figure 1). Four levels of box weights were evaluated in each mode: no-load (i.e., unloaded, empty-handed reference condition), 4.5 kg, 9.1 kg, and 13.6 kg. The load levels were 20%, 40%, and 60%, respectively, of the maximum permissible lifting load of 23 kg specified by the NIOSH Lifting Equation [49]. Magnitudes and distances were informed by previous field-based studies on MMH in industrial settings [7,8] and laboratory studies on manual load carriage, e.g., [3,22,50]. The two-handed side load carriage was performed using a box in each hand with a handle on the top (152.4 mm width × 177.8 mm depth × 127 mm height, Figure 1a). The two-handed anterior carry was performed using one box with two handles on the sides (177.8 mm width × 228.6 mm depth × 203.3 mm height, Figure 1b). To maintain symmetry in hand-loads, the combined weight in the anterior carry was equally divided between the right and left hand in the two-handed side carry.

Two walk trials in the no-load condition were performed at the beginning of the experiment. Subsequently, each participant performed two consecutive walk trials with hand loads in each combination of carrying mode and load level, presented in random order to minimize any potential influence of cumulative fatigue on gait kinematics. Participants were instructed to self-select a walking speed for each condition that could be comfortably maintained over the two consecutive walk trials. In the two-handed anterior condition, participants were instructed to hold the box close to their torso with their elbow flexed 90${}^{\circ }$. In order to minimize carryover effects of fatigue, participants were provided a two-minute rest break between each condition and were allowed additional rest breaks anytime if they requested.

#### 2.1.3. Instrumentation

During the walk trials, kinematic data were continuously recorded using four commercial inertial sensors, namely, Opal (APDM Inc., Portland, OR, USA; dimension = 43.7 × 39.7 × 13.7 mm (L × W × H); weight = 25 g; internal storage = 8 GB; https://www.apdm.com/). Each sensor is comprised of a 3-axis accelerometer (±16 g), a 3-axis gyroscope (±2000 ${}^{\circ }$/s), and a 3-axis magnetometer (±8 Gauss).

Two sensors were attached using elastic Velcro straps over the sixth thoracic vertebra (T6) and the first sacral vertebra (S1), respectively (Figure 1). One sensor was positioned along the superior aspect of the right thigh midway between the hip and lateral femoral epicondyles, and the fourth sensor on the superior aspect of the right shank midway between the lateral femoral and malleolar epicondyles, and attached using double-sided hypoallergenic tape and medical tape wrap. Sensors were attached with one of the sensor axes (i.e., *x*-axis) aligned with the proximal-distal axis of the body segment and pointing inferiorly.

#### 2.1.4. Data Processing and Dependent Measures

Only gyroscope data were utilized in this study. The four inertial sensors synchronously recorded triaxial gyroscope data at a sampling frequency of 80 Hz. Each walk trial lasted no more than 30 s. Raw gyroscope data for each walk trial were filtered using a second-order low-pass zero-lag Butterworth filter with a cut-off frequency of 2 Hz.

Next, a custom software algorithm implemented in MATLAB (MATLAB R2016b, The MathWorks Inc., Natick, MA, USA) was used for computing particular spatio-temporal gait parameters. Specifically, gait cycle duration and stride length were computed in order to segment the continuous sensor data into discrete gait cycles. Filtered data were integrated to obtain angular displacement, and subsequently filtered using a second-order high-pass filter with a cut-off frequency of 0.75 Hz to reduce the effect of drift [51]. Torso (T6) and pelvic (S1) sway and their corresponding thoracic-pelvic coordination in the form of relative phase angles in the coronal, transverse, and sagittal planes respectively were computed from the gyroscope data for each segmented gait cycle. Table 1 provides definitions and sensor locations used for computing these 11 parameters with the computational procedure briefly described below.

*Spatio-temporal parameters.* First, heel-strike (i.e., when the foot first touches the floor) and toe-off events were detected using the filtered angular velocity data (sagittal plane) obtained from the gyroscope on the right shank using an algorithm adapted from Aminian et al. [42] and validated in multiple prior studies [52,53]. Time duration between two consecutive right heel-strikes were denoted as one gait cycle, which is summarized in Appendix A. In a study to validate the algorithm by comparing gyroscope-derived gait events to corresponding measurement from foot pressure sensors, Aminian et al. [42] reported no statistically significant error for toe-off detection, and an average delay of 10 ms for detecting heel-strike events.

Stride length was estimated using the double segment gait model by Aminian et al. [42] and summarized in Appendix B. The double segment gait model estimates stride length by considering the thigh and shank as a double pendulum during the swing phase, and likewise as an inverted double pendulum during the stance phase with the assumption of symmetry between both legs [42]. A previous validation of this algorithm reported RMSE’s of 23 ms for gait cycle duration, and 7 cm (7.2%) for stride length compared to reference gait data obtained from foot pressure sensors [42].

*Torso and Pelvis Postural Sway.* For each gait cycle, six measures of torso and pelvic sway were calculated as the peak-to-peak range of motion (ROM) angles in the coronal, transverse, and sagittal planes at the thoracic (sensor at T6) and pelvic (sensor at S1) segment location, respectively. The angular displacements were calculated by integrating the filtered gyroscope data within each gait cycle (~1.0 s average duration).

*Thoracic-Pelvic Coordination.* Thoracic-pelvic coordination was measured in each of the three anatomical planes using the mean relative phase angle [34] computed at each data-frame and averaged over each gait cycle (refer Appendix C). A higher mean relative phase angle implied an out-of-phase or uncoordinated movement between the thorax and pelvis segments compared to more in-phase (i.e., synchronized and coordinated movements) for lower mean relative phase angles.

#### 2.1.5. Statistical Analyses

Five gait cycles were extracted from each walk trial for analysis. The 2nd to 6th gait cycle were used in order to minimize the effects of acceleration and deceleration near the start and end of the walk trials. Eleven gait parameters were computed for a total of 720 gait cycles (i.e., 9 participants × 8 conditions × 2 repetitions per condition × 5 gait cycles per walk trial). Computed gait parameters were averaged over five gait cycles within each walk trial for subsequent statistical analyses.

Statistical analyses were conducted in two stages. First, descriptive statistics and Pearson correlation coefficients for the nine thoracic-pelvic sway and coordination parameters over two walk trials each averaged over five gait cycles per condition were calculated. Second, separate mixed effects models [54] implemented in SPSS v.26 (IBM Inc., New York, NY, USA) were used to quantify the effects of within-subject variables, viz., carrying mode (two-handed side carry vs. two-handed anterior carry) and load level (no-load, 4.5 kg, 9.1 kg, and 13.6 kg) and their two-way interaction effect on each of the nine gait parameters. Self-selected walking speed was a potential confounding variable since it influences gait posture, and is known to be influenced by leg length (or stature) [55] as well as external load conditions [56]. Relative speed (walking speed/leg length; m/s) is commonly used in gait studies as a measure of walking speed (stride length/gait cycle duration, m/s) normalized to individual anthropometry, typically leg length or stature (e.g., [57,58,59]). Thus, the centered value of relative walking speed (labeled ’centered relative speed’) was included in each mixed effects model as a covariate (i.e., set as a fixed effect and random effect parameter) to adjust for differences in anthropometry and walking speed. Two walk trials in each condition were included as repeated measures. Residual errors from the mixed model estimates were examined graphically and confirmed that model assumptions of normality (Q-Q plots) and homogeneity of variances (box-plots of residuals across carrying mode and load levels) were satisfied. Significant main and interaction effects (p<0.05) were examined using Bonferroni post-hoc tests which adjusts for Type-I error rates to analyze paired comparisons between carrying mode at each level of load level and vice versa. Estimated marginal means and standard errors (±SE) for the 9 gait parameters obtained from the mixed effects models were tabulated, stratified by carrying mode and load level, at the average values of centered relative speed.

### 2.2. Results

The nine participants had an average (±standard deviation, SD) age of 21.7 ± 2.7 years (range: 18–27 years), stature 1.76 ± 0.06 m (range: 1.64–1.84 m), leg length 0.94 ± 0.06 m (range: 0.84–1.04 m), body mass 77.47 ± 10.64 kg (range: 64.95–93.26 kg), and BMI 24.87 ± 2.84 kg/m^2^ (range: 20.99–30.36 kg/m^2^). Average relative speed across all gait cycles were 1.09 ± 0.08 m/s (range: 0.96–1.24 m/s). Correlation coefficients between pairs of sway and coordination parameters were below 0.51.

Table 2 summarizes the results from the mixed effects analyses of carrying mode and load level on thoracic and pelvic sway and coordination. Table 3 provides the estimated marginal means (±SE) for these parameters with statistically significant (p<0.05) effects of carrying mode and load level relative to the no-load condition, at average values of the covariate, centered relative speed. Significant trends across carrying mode and load level are discussed in the subsequent section.

#### 2.2.1. Torso and Pelvis Sway and Coordination in the Coronal Plane

**Figure 2:**
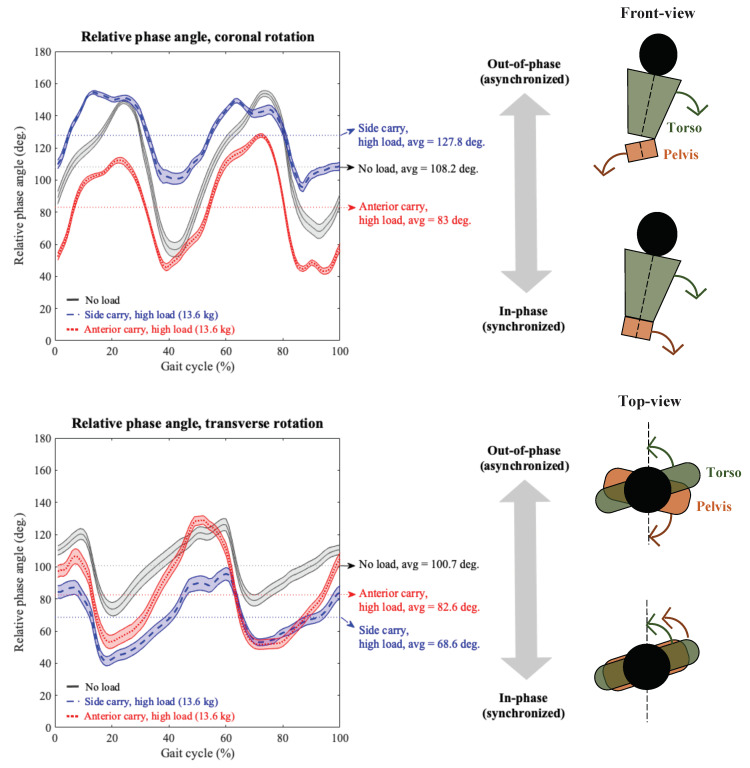
Mean (±standard error) thoracic-pelvic relative phase angle in the coronal plane (**top** panel) and transverse plane (**bottom** panel) obtained from the average gait cycle data of *n* = 9 participants and normalized to individual gait cycle duration. The figure compares two-handed side carry and two-handed anterior carry (colored blue and red, respectively) in high load level at 13.6 kg with respect to the no-load (empty-handed; grey). Higher values of mean relative phase angles indicate out-of-phase or less synchronized rotational movements between the torso and pelvis.

In the coronal plane, thoracic sway was significantly more in the no-load and low load levels vs. medium, and high load conditions (Table 2), while pelvic sway did not indicate any significant differences between carrying mode and load level. A significant interaction effect of carrying mode and load on mean coronal relative phase angle was obtained. Coronal relative phase angle was significantly less in the anterior carry vs. side carry mode for the low, medium and high load conditions separately, suggesting more in-phase thoracic-pelvic movement in the anterior vs. side carry condition (Figure 2). In the side carry condition, the mean relative phase angle was significantly more in the high load condition vs. the medium load, low load, and no-load conditions. Conversely, in the anterior carry condition, the mean coronal relative phase angle was significantly higher in the no-load condition compared to each of the loaded conditions.

#### 2.2.2. Torso and Pelvis Sway and Coordination in the Transverse Plane

In the transverse plane, thoracic rotation was not significantly different across carrying mode and load level. However, transverse rotation at the pelvis was significantly more in the side carry compared to the anterior carry. The mean relative phase angle in the transverse plane was significantly less in the side carry vs. anterior carry. The mean relative phase angle in the transverse plane was significantly less in all of the loaded conditions compared to the unloaded condition.

#### 2.2.3. Torso and Pelvis Sway and Coordination in the Sagittal Plane

A significant interaction effect of carrying mode and load level on thoracic rotation in the sagittal plane was obtained. Thoracic rotation was significantly more in the side carry vs. anterior carry mode for the low, medium, and high load conditions separately. Pelvic sagittal rotation was not significantly different among carrying modes and load levels. Mean sagittal relative phase angle was significantly more in the side carry vs. anterior carry mode.

## 3. Experiment 2

### 3.1. Materials and Methods

#### 3.1.1. Participants

Based on promising results obtained from Experiment-1, a second experiment was conducted. Participants for Experiment-2 consisted of ten healthy male individuals aged between 18 to 55 years old different from Experiment-1. Compared to the previous experiment, the age range for participant recruitment was expanded to diversify the sample population. Participants were screened for pre-existing back injuries or chronic pain and provided informed consent as in Experiment 1 (Section 2.1.1).

#### 3.1.2. Experiment Procedure

Experiment conditions and data collections procedures were the same as in Experiment 1 (described in Section 2.1.2). The experiment had participants carry a weighted box in a straight, marked path (12 m length × 1.6 m width) on a levelled floor for a distance of 10 m.

#### 3.1.3. Instrumentation

Experiment 2 used a different commercial wearable inertial sensor (Biostamp RC; mc10 Inc., Lexington, MA, USA; dimension = 66 × 34 × 4.5 mm (L × W × H); weight = 7 g; internal storage = 32 MB; https://www.mc10inc.com/), which consists of a flexible and conformal skin-adhesive patch with an embedded 3-axis accelerometer (±16 g) and 3-axis gyroscope (±2000 ${}^{\circ }$/s) sensor. The Biostamp RC was considered because of its comparable accuracy (e.g., [52,61]) and improved wearability relative to the other commercial IMUs that have a hard enclosure and elaborate attachment process. The device is also 510(k) cleared by FDA for medical use.

Prior to Experiment 2, a simple empirical validation of the two sensors, namely APDM Opal (Expt-1) and BioStampRC (Expt-2) was performed to ensure the accuracy of their gyroscope-based angular displacement estimates relative to an optical motion capture (Qualisys AB, Gotenburg, Sweden) based estimate as reference. A simple pendulum consisting of a non-ferrous rigid arm (arm length: 460 mm; mass: 0.275 kg) was used since our study modelled the torso and pelvis as segments of an oscillating inverted pendulum.

For validation, two passive optical markers (used for computing the reference angular displacement) along wth an APDM Opal and Biostamp RC sensor were attached to the pendulum arm. Starting from an initial angular displacement of approximately 75${}^{\circ }$, continuous oscillations were recorded for 30 s durations (i.e., considered representative of a walk trial duration). For time synchronization between different measurement systems, the pendulum arm was moved at a fast speed to create identifiable peaks before the start of the pendulum movement. Time-synchronized 2D angular displacement computed from the two gyroscope systems and passive optical markers from three trials of 30 s duration each were compared. The same filtering methods were used for both gyroscope and optical motion marker data. The average ± SD RMSE between both gyroscopes was 0.22 ± 0.09${}^{\circ }$, while the RMSE between the optical motion capture and Opal and Biostamp RC was 0.53 ± 0.06${}^{\circ }$ and 0.31 ± 0.03${}^{\circ }$, respectively. The relatively low RMSE (<1${}^{\circ }$) between instrumentation types was deemed acceptable for the purposes of comparing 2D angular displacement data from the respective sensors used in Experiments 1 and 2. For the instrumented walk trials, four sensors were attached on the participant at identical anatomical locations to Experiment-1 using the manufacturer-provided adhesive tape in order to continuously record kinematic data during the walk trials. Sensor setup was the same as Experiment 1 as described in Figure 1.

#### 3.1.4. Data Processing, Dependent Measures, and Statistical Analysis

Data obtained from the experiment walk trials were processed in a manner identical to Experiment 1 (Section 2.1.4). Eleven gait parameters were computed (Table 1) for a total of 797 gait cycles (i.e., 10 participants × 8 conditions × 10 gait cycles per condition minus the 6th gait cycle from three different walk trials discarded due to measurement issues). Statistical analyses identical to Experiment-1 (Section 2.1.5) were performed.

### 3.2. Results

The ten participants had an average ± SD age of 38.6 ± 10.8 years (range: 22–55 years), stature 1.81 ± 0.05 m (range: 1.72–1.88 m), leg length 0.96 ± 0.05 m (range: 0.88–1.03 m), body mass 80.75 ± 12.20 kg (range: 57.60–96.60 kg), and BMI 24.70 ± 3.17 kg/m^2^ (range: 19.47–28.85 kg/m^2^). Average relative speed across all gait cycles were 1.01 ± 0.15 m/s (range: 0.83–1.25 m/s). Correlation coefficients between pairs of sway and coordination parameters were below 0.75.

Table 4 summarizes results from the mixed effects analyses of carrying mode and load level on torso and pelvic sway and coordination parameters. Table 5 provides the estimated marginal means (± SE) for these nine parameters with statistically significant (p<0.05) effects of carrying mode and load level relative to the no-load condition, estimated at the average value of centered relative speed (covariate).

#### 3.2.1. Torso and Pelvis Sway and Coordination in the Coronal Plane

In the coronal plane, thoracic sway was significantly more in the no-load vs. the medium and high load conditions, and also significantly more in the low vs. medium load condition. Pelvic sway was significantly more in the side vs. anterior carry mode. A signficant interaction effect of carrying mode and load level on mean coronal relative phase angle was obtained. Coronal relative phase angle was significantly less in the anterior carry vs. side carry mode for the low, medium, and high load conditions separately, suggesting more in-phase thoracic-pelvic movement in the anterior vs. side carry condition akin to Experiment 1 (Figure 2). In the anterior carry mode, the mean relative phase angle was significantly more in the no-load condition compared to the high load condition.

#### 3.2.2. Torso and Pelvis Sway and Coordination in the Transverse Plane

In the transverse plane, thoracic sway was significantly more in the side carry compared to the anterior carry mode. Pelvic sway was significantly more in the side carry vs. anterior carry mode in each of the three loaded conditions separately. The mean relative phase angle in the transverse plane was significantly less in medium and high load vs. unloaded conditions, regardless of carrying mode.

#### 3.2.3. Torso and Pelvis Sway and Coordination in the Sagittal Plane

In the sagittal plane, a significant interaction effect of carrying mode and load level on thoracic sway was noted. Thoracic sway was significantly more in the side carry vs. anterior carry mode in the low, medium and high load conditions separately. Furthermore, in the side carry, the high and medium load conditions showed significantly more thoracic sway compared to the no-load condition. Pelvic sagittal rotation and mean sagittal relative phase angle were not significantly different between different carrying modes and load levels.

## 4. General Discussion

The purpose of this study was to quantify the effects of hand loads during two-handed side and anterior load carriage on thoracic-pelvic sway and coordination obtained from body-worn gyroscopes while walking at a self-selected speed. By including relative speed as a covariate, the study estimated the marginal effects of carrying mode and load level after adjusting for individual anthropometry (leg length) and preferred walking speed, which are two known variables that affect gait kinematics. Results from two separate experiments demonstrate that two-handed side and anterior load carriage relative to unloaded walking are associated with systematic influences on thoracic-pelvic sway and coordination. The two experiments differed in terms of their anthropometric diversity, namely, relatively homogenous (Expt-1) and age-diverse (Expt-2) male cohorts, and the choice of commercial wearable sensor, yet produced convergent findings suggesting consistency in findings.

Prior to discussing the study’s key findings, it is worth noting that studies have previously investigated biomechanical adaptations in gait due to the magnitude and position of hand loads [2,26,27,29,30,33]. However, these studies primarily involved either walking on treadmills at controlled speeds or on ground without accounting for walking speed. Moreover, these studies examined diverse carrying modes and magnitudes of load including a fixed amount, or loads normalized to percentage of body weight. None of the reviewed studies characterized the specific measures of thoracic-pelvic coordination (i.e., relative phase angles) in two-handed anterior and/or side carry. Studies that investigated thoracic-pelvic or lumbar-sacral coordination used other modes of carrying (e.g., backpacks [25], overhead firearm carry [35]), while studies on anterior and/or side carry examined other types of kinematic and biomechanical measures [3,20,38,50]. As a result, direct comparisons with the present study are not possible. However, the direction of change in gait and postural kinematics observed in the present study are supported by findings from prior studies and are briefly discussed here.

In general, the peak-to-peak magnitude changes in thoracic and pelvic sway were small and on their own were of potentially less practical value. More notable was the combining of thoracic and pelvic sway over the entire gait cycle into a normalized measure of thoracic-pelvic coordination, i.e., relative phase angle, which yielded distinct and meaningful trends primarily in the coronal and transverse planes. In both experiments, side load carriage was characterized by an increase in pelvic sway in the transverse plane and an increase in thoracic sway in the sagittal plane compared to anterior load carriage. Regarding thoracic-pelvic coordination in the coronal plane, both experiments indicated a significant interaction effect of carrying mode and load. Thoracic-pelvic coordination in the *coronal* plane was more asynchronous or out-of-phase in the side vs. anterior carry, and these differences were more pronounced at higher load levels, i.e., coronal movement was more asynchronous in side carry and more coordinated in anterior carry at the high vs. low load levels. In general, thoracic-pelvic movement in the *transverse* plane was significantly more coordinated in the loaded vs. unloaded walk conditions, which is a trend also reported in other modes of load carriage [25,35]. Comparing between modes, thoracic-pelvic movement was more coordinated in the side vs. anterior carry, though statistically significant only in Experiment-1. Thoracic-pelvic movement in the *sagittal* plane was slightly more coordinated in the anterior carry vs. side carry and significant only in Experiment-1, but these differences were smaller compared to the effects on thoracic-pelvic coordination in the coronal and transverse planes. It is possible that the relatively small sample size relative to the sample diversity may have contributed to reduced statistical power to detect certain differences in Experiment-2. These trends in postural sway and thoracic-pelvic coordination across carrying modes and load magnitudes can be explained as follows by changes in body dynamics [25,29], specifically, angular momentum and location of the combined center of mass (COM) in response to the body’s need for maintaining dynamic balance while walking/carrying.

### 4.1. Analysis of Two-Handed Side Carry

During the swing phase of walking, the upper torso including the arms counter-rotate relative to the pelvis in the transverse plane to reduce net angular momentum [62]. In a side carry with the external load equally divided between the right and left hands, there is a net increase in the moment of inertia and the angular momentum in the transverse plane from loads located laterally [50]. Consequently, the angular momentum of the rest of the body needs to increase in the opposite direction to reduce the net angular momentum. This increase is accomplished by synchronizing the transverse rotation of the torso and pelvic segments and evinced as increased pelvic sway and more in-phase coordination between thoracic-pelvic segments in the transverse plane relative to unloaded gait.

Hand loads in a side carry lower the location of the COM of the upper body in the coronal and the sagittal planes. Consequently, the moment of inertia and angular momentum of the torso in the coronal plane increases. With the increasing hand loads, angular momentum of the upper body in the coronal plane is controlled by limiting thoracic coronal sway. Pelvic sway in the coronal plane was found to increase with increasing load levels to compensate for this reduced rotation of the torso. Furthermore, the torso and pelvis tend to counter-rotate in the coronal plane in order to decrease the net angular momentum between the torso and pelvis, which was evident from the more out-of-phase coordination between thoracic-pelvic segments with increasing load levels.

### 4.2. Analysis of Two-Handed Anterior Carry

Rotation of the arms is restricted in a two-handed anterior carry since the torso, arms, and hand load are coupled and move together [3,35,63]. In order to reduce the net angular momentum in the transverse plane while walking, the upper body including the arms and the trunk counter-rotate relative to the lower body. The need for increased postural stability due the anterior load is achieved by co-contracting the trunk muscles and limiting rotational movement in the upper body [19] and by a concomitant decrease in pelvic sway in the transverse plane with increasing anterior loads. This decreased transverse pelvic rotation during load carriage is compensated by a simultaneous increase in hip excursion [25] and reflected in the increased pelvic sway recorded in the sagittal plane.

During anterior load carriage, coordination between the thoracic-pelvic segments was more in-phase or synchronized in the loaded conditions vs. no-load in the coronal and transverse planes indicating the close coupling between the torso and pelvis. Since the load is located medially in the transverse and coronal planes during anterior load carriage in contrast to the laterally positioned loads in a side carry, the moment of inertia and angular momentum in the coronal plane at the torso is less in the anterior carry [50]. Thus, the need for counter-rotating the upper vs. lower body to reduce the net angular momentum between the segments is relatively lower compared to the side carry. This difference in angular momentum and trunk movements between modes produced diverging trends in thoracic-pelvic coordination in the anterior carry (i.e., more in-phase) compared to the side carry (i.e., more out-of-phase) with increasing load magnitude.

### 4.3. Study Contributions and Limitations

Collectively, the findings from this study help typify naturalistic postural adaptations to load carriage under conditions simulating manual material handling that commonly occur in the workplace. The application of wearable inertial sensors, including gyroscopes, accelerometers, and IMUs enables the study of such ambulatory work tasks. The present study specifically focused on a minimal set of body-worn gyroscope sensors (i.e., two for thoracic-pelvic coordination, and two for spatiotemporal gait paramaters) as a prelude to subsequent studies in applied work settings. Prior studies on posture and gait during load carriage have typically used optical motion tracking in controlled laboratory environments, often while walking on a treadmill at a precise speed. The present study had walk trials performed in more naturalistic conditions with participants walking at a self-selected speed in a sufficiently long and levelled corridor. This was done in order to record natural adaptations in gait patterns associated with the carrying mode and load level without imposing any external constraint on gait speed. However, this implied having to adjust for potential confounding variables related to individual anthropometry and gait. Earlier studies support the notion that walking speeds and stride length are slightly modified depending on the loads carried [25,30,64]. The present study used a normalized measured of walking speed, namely relative speed, obtained from gyroscope-based estimates of gait cycle duration and stride length as a covariate to account for between and within-participant differences in walking speed and stature. The covariate was statistically significant in nearly all of the dependent measures tested in coronal and transverse planes, justifying its inclusion. Despite this statistical treatment, it is possible that some of the postural parameters were influenced by differences in anthropometry and/or strength (e.g., hip abduction strength) factors not measured in this study besides load intensity and carrying mode.

Only two carrying modes were considered in this study. A shift in the center of mass posteriorly such as while wearing a backpack, e.g., [14,22,25,28,33], or laterally with a load in one hand or on one shoulder, e.g., [17,19,20,22,33], may result in gait adaptations different from those produced in this study. However, our findings on thoracic-pelvic sway and coordination during the anterior carry were similar to findings on the effects of posterior load carriage reported by [25] wherein a loaded backpack carry decreased transverse thoracic and pelvic rotation, and the mean relative phase angle between thoracic and pelvic segments, suggesting similar effects on gait kinematics. The load magnitudes used in the present study were lower than some previous studies (e.g., 40% of the body weight [25]) and focused on multiple relatively short bouts of walking (<30 s per trial) to reflect load magnitudes and carrying distances found in the workplace (e.g., between workstations, [7,8]). With heavier loads, longer walking durations, and onset of whole-body fatigue, the metabolic cost of load carriage is expected to increase causing even further alterations in muscle activation patterns, posture, and gait kinematics [65,66]. The present study enforced two-minute rest breaks between walk trials to minimize the cumulative effects of fatigue. Based on walking speeds obtained in Experiment-1, the path length in Experiment-2 was reduced from 26.2 m to 12 m to further minimize possible effects of cumulative fatigue, while ensuring that five cycles of steady-state gait per trial (i.e., 2nd to 6th gait cycle) could be obtained in a consistent manner.

As an initial investigation, the present study was limited to an age-diverse cohort of male participants with multiple gait cycles analyzed per condition and participant, resulting in 1517 gait cycles analyzed. Subsequent studies would need to consider diversity in functional strength and gender among other known sources of variability in gait. In both experiments (Expt-1 with a homogeneous sample & Expt-2 with a diverse sample), pelvic-thoracic coordination in coronal and transverse planes indicated significant and plausible differences in carrying mode and magnitude of load suggesting consistent kinematic adaptations. However, the relatively small sample size causes both experiments to have low statistical power. Hence, certain statistical differences may possibly go undetected, for example, the absence of a significant effect of carrying mode on sagittal thoracic-pelvic coordination in Experiment-2 (compared to Experiment-1). Nevertheless, the relatively healthy, all-male cohort in these initial studies help establish baseline expectations for thoracic-pelvic coordination patterns during load carriage, and facilitates reference comparisons in future studies investigating effects and/or onset of physical fatigue, or presence of work-related disorders such as low-back pain e.g., [67], on dynamic gait posture during load carriage.

The findings from the present study suggest that information about both carrying mode and load magnitude can be obtained from a combination of thoracic-pelvic sway and coordination. These findings have potential for informing approaches to assessing exposures from load carriage [68] in work settings where the intensity, duration, frequency, and mode of load carriage varies over time, such as in construction work, warehousing, packaging, and distribution. Field-based studies of biomechanical exposure assessment typically require instrumentation for force measurement such as surface electromyography for muscle activation levels and force platform for measuring ground reaction forces at the foot that is conspicuous, cumbersome, and potentially interferes with workers movements and task performance. Instead, wearable sensing technologies such as gyroscopes and IMUs could provide a low-cost and less obtrusive alternative for use in field-based studies. However, certain algorithmic limitations such as related to sensor drift have yet to be overcome. The present study circumvents issues of drift by integrating angular velocity data only within each gait cycle for computing stride length, thoracic-pelvic sway, and coordination (i.e., typically less than 1 s duration).

The present study was limited to short duration walk trials, and used a previously validated algorithm that used gyroscope data for gait detection. While algorithmic approaches to detecting gait events from body-worn gyroscopes and IMUs have received considerable attention in the biomechanics and clinical literature, e.g., [42,69,70,71,72], further studies are needed to identify and validate those sensor configurations and algorithms in real-world environment (e.g., [73]) and occupational settings (e.g., uneven or different terrain types in construction sites, e.g., [74]). In addition, the growing interest of applying machine learning algorithms to extract contextual information beyond postural angles such as the type of work tasks and/or load intensities in the context of MMH using body-worn inertial sensor data can broaden the use of wearable sensors for field-based assessment of biomechanical exposures and associated injury risk [68,75,76]. An important step when constructing such machine learning algorithms is the selection of relevant and plausible features that can accurately and reliably discriminate among task conditions. Findings from the present study suggest thoracic-pelvic coordination as candidate features for such machine learning algorithms.

## 5. Conclusions

Thoracic-pelvic coordination when walking is challenged by the addition of external hand loads. This study identified distinct patterns in thoracic-pelvic coordination from different hand loads in two-handed side vs. anterior load carriage at self-selected walking speeds using body-worn gyroscopes, after adjusting for relative speed. These differences reflect postural adjustments to maintain dynamic postural stability while walking under the external load conditions. Rotational movement coordination between the torso and pelvis measured as relative phase angles in the coronal and transverse planes were particularly insightful to quantify the effects of hand-load magnitude and carrying mode on walking. Study findings suggest that relative phase angles measured in the coronal and transverse planes may provide sufficiently sensitive information to characterize changes in gait posture induced by carrying mode and the magnitude of hand-load. These findings serve as a foundation for future studies using wearable sensing in applied work settings to measure posture adaptations and effects of exposure to biomechanical stress from manual load carriage.

## Figures and Tables

**Table 1:** List and definitions of gait parameters calculated from inertial sensor data. In each row, location of sensors used for calculating the parameters are indicated by ’•’ with relevant source reference. Connected dots indicate pairs of sensors used together.

Parameter		Definition	Inertial Sensor Location				Source
			T6	S1	R. Thigh	R. Shank	
**Spatio-temporal parameters**		
1	Gait cycle duration (sec)	The duration of one gait cycle (one right plus left step duration calculated as the time between two consecutive right heel-strikes)				•	[42]
2	Stride length (cm)	The length moved from right heel-strike to the next right heel-strike during one gait cycle			•	•	[42]
**Torso and pelvis postural sway**		
3, 4	Coronal ROM at T6 & S1 (${}^{\circ }$)	Range of rotation angle in coronal plane: Max (integrated angular velocity, *z*-axis) - min (integrated angular velocity, *z*-axis)	•	•			[25]
5, 6	Transverse ROM at T6 & S1 (${}^{\circ }$)	Same calculation as above in transverse plane: *x*-axis	•	•			
7, 8	Sagittal ROM at T6 & S1 (${}^{\circ }$)	Same calculation as above in sagittal plane: *y*-axis	•	•			
**Thoracic-pelvic coordination**		
9	Coronal mean relative phase angle between T6 and S1 (${}^{\circ }$)	Average (pelvic phase angle - thoracic phase angle). Phase angle (t) = arctan (normalized angular velocity, *z*-axis (t)/normalized integrated angular velocity, *z*-axis (t))	•——•				[25,34,60]
10	Transverse mean relative phase angle between T6 and S1 (${}^{\circ }$)	Same calculation as above in transverse plane: *x*-axis	•——•				
11	Sagittal mean relative phase angle between T6 and S1 (${}^{\circ }$)	Same calculation as above in sagittal plane: *y*-axis	•——•				

**Table 2:** Experiment-1 (*n* = 9) summary results from the mixed effects analyses for the main and interaction effects of carrying mode and load level on torso and pelvis sway and thoracic-pelvic coordination measures in the coronal, transverse, and sagittal planes. Significant pair-wise Bonferroni comparisons (*p* < 0.05) are provided for main and interaction effects that were significant at *p* < 0.05 indicated by *. NL = No-load (empty-handed), L = Low load at 4.5 kg, M = Medium load at 9.1 kg, H = High load at 13.6 kg.

	Carrying Mode	Load Level	Carrying Mode × Load Level	Relative Speed, Centered (s)
**Coronal plane**	
ROM at T6 (${}^{\circ }$)	*F*(1, 114) = 0.24, *p* = 0.623	*F*(3, 53) = 11.89, *p* < 0.001 *	*F*(3,53) = 0.15, *p* = 0.930	*F*(1, 106) = 112.67, *p* < 0.001 *
		(NL, L) > (M, H)		
ROM at S1 (${}^{\circ }$)	*F*(1, 130) = 0.02, *p* = 0.891	*F*(3, 66) = 2.52, *p* = 0.066	*F*(3, 66) = 0.11, *p* = 0.952	*F*(1, 113) = 8.05, *p* = 0.005 *
Mean relative phase angle (${}^{\circ }$)	*F*(1, 101) = 96.96, *p* < 0.001 *	*F*(3, 71) = 1.72, *p* = 0.170	*F*(3, 72) = 15.35, *p* < 0.001 *	*F*(1, 95) = 39.03, *p* < 0.001 *
	Side > Anterior		Side: H > (M, L, NL)	
			Anterior: NL > (L, M, H)	
			H, M, L: Side > Anterior	
**Transverse plane**	
ROM at T6 (${}^{\circ }$)	*F*(1, 118) = 1.65, *p* = 0.202	*F*(3, 55) = 0.61, *p* = 0.612	*F*(3, 55) = 1.17, *p* = 0.330	*F*(1, 1) = 18.46, *p* = 1.0
ROM at S1 (${}^{\circ }$)	*F*(1, 101) = 7.41, *p* = 0.008 *	*F*(3, 63) = 1.33, *p* = 0.272	*F*(3, 63) = 1.47, *p* = 0.231	*F*(1, 95) = 7.03, *p* = 0.009 *
	Side > Anterior			
Mean relative phase angle (${}^{\circ }$)	*F*(1, 128) = 8.59, *p* = 0.004 *	*F*(3, 62) = 4.98, *p* = 0.004 *	*F*(3, 62) = 1.07, *p* = 0.371	*F*(1, 110) = 0.04, *p* = 0.842
	Anterior > Side	(NL, L) > (M, H)		
**Sagittal plane**	
ROM at T6 (${}^{\circ }$)	*F*(1, 113) = 28.55, *p* < 0.001 *	*F*(3, 60) = 0.58, *p* = 0.630	*F*(3, 60) = 3.11, *p* = 0.033 *	*F*(1, 91) = 0.08, *p* = 0.775
	Side > Anterior		H, M, L: Side > Anterior	
ROM at S1 (${}^{\circ }$)	*F*(1, 128) = 2.95, *p* = 0.088	*F*(3, 65) = 2.07, *p* = 0.113	*F*(3, 65) = 0.69, *p* = 0.565	*F*(1, 1) = 16.35, *p* = 1.0
Mean relative phase angle (${}^{\circ }$)	*F*(1, 122) = 7.14, *p* = 0.009 *	*F*(3, 57) = 0.53, *p* = 0.664	*F*(3, 57) = 1.30, *p* = 0.284	*F*(1, 103) = 7.24, *p* = 0.008 *
	Side > Anterior			

**Table 3:** Experiment-1 (*n* = 9) estimated marginal mean ± SE for the No-load condition, and statistically significant mean differences ± SE relative to the no-load condition by carrying mode and load level, at average values of centered relative speed. Significant mean differences relative to the no-load condition by carrying mode across different load levels are within the square brackets (i.e., [- - mean ± SE - -]).

		Side Carry			Anterior Carry		
	No-Load	Low (4.5 kg)	Medium (9.1 kg)	High (13.6 kg)	Low (4.5 kg)	Medium (9.1 kg)	High (13.6 kg)
**Coronal plane**							
ROM at T6 (${}^{\circ }$)	5.6 ± 0.3	-	−1.7 ± 0.3	−1.8 ± 0.4	-	−1.7 ± 0.3	−1.8 ± 0.4
ROM at S1 (${}^{\circ }$)	7.5 ± 0.4	-	-	-	-	-	-
Mean relative phase angle (${}^{\circ }$)	109.5 ± 5.2	+5.0 ± 7.0	+8.4 ± 6.0	+23.6 ± 5.9	−22.2 ± 6.0	−22.9 ± 6.5	−30.2 ± 6.1
**Transverse plane**							
ROM at T6 (${}^{\circ }$)	5.6 ± 0.4	-	-	-	-	-	-
ROM at S1 (${}^{\circ }$)	7.4 ± 0.4	[ - - - - - - - - - - - - +1.7 ± 0.6 - - - - - - - - - - - - - - ]			-	-	-
Mean relative phase angle (${}^{\circ }$)	102.2 ± 7.6	−30.5 ± 10.7	−30.4 ± 10.5	−33.8 ± 9.1	-	−11.6 ± 10.3	−18.8 ± 10.6
**Sagittal plane**							
ROM at T6 (${}^{\circ }$)	2.9 ± 0.2	+0.5 ± 0.3	+0.5 ± 0.3	+0.6 ± 0.3	−0.2 ± 0.2	−0.5 ± 0.2	−0.3 ± 0.2
ROM at S1 (${}^{\circ }$)	3.8 ± 0.7	-	-	-	-	-	-
Mean relative phase angle (${}^{\circ }$)	81.7 ± 3.5	[ - - - - - - - - - - - - +1.8 ± 4.5 - - - - - - - - - - - - - - ]			[ - - - - - - - - - - - - - −9.0 ± 4.5 - - - - - - - - - - - - - - ]		

**Table 4:** Experiment-2 (*n* = 10) summary results from the mixed effects analyses for the main and interaction effects of carrying mode and load level on torso and pelvis sway and thoracic-pelvic coordination in the coronal, transverse, and sagittal planes. Significant pairwise Bonferroni comparisons (*p* < 0.05) are provided for main and interaction effects that were significant at *p* < 0.05 indicated by *. NL = No-load (empty-handed), L = Low load at 4.5 kg, M = Medium load at 9.1 kg, H = High load at 13.6 kg.

	Carrying Mode	Load Level	Carrying Mode × Load Level	Relative Speed, Centered (s)
**Coronal plane**	
ROM at T6 (${}^{\circ }$)	*F*(1, 123) = 0.02, *p* = 0.896	*F*(3, 79) = 6.48, *p* = 0.001 *	*F*(3, 78) = 0.69, *p* = 0.558	*F*(1, 93) = 48.81, *p* < 0.001 *
		NL > (M, H), L > M		
ROM at S1 (${}^{\circ }$)	*F*(1, 149) = 5.16, *p* = 0.025 *	*F*(3, 72) = 0.91, *p* = 0.442	*F*(3, 71) = 0.71, *p* = 0.548	*F*(1, 124) = 24.91, *p* < 0.001 *
	Side > Anterior			
Mean relative phase angle (${}^{\circ }$)	*F*(1, 125) = 28.52, *p* < 0.001 *	*F*(3, 76) = 1.12, *p* = 0.346	*F*(3, 75) = 3.91, *p* = 0.012 *	*F*(1, 122) = 5.01, *p* = 0.027 *
	Side > Anterior		Anterior: NL > H	
			H, M, L: Side > Anterior	
**Transverse plane**				
ROM at T6 (${}^{\circ }$)	*F*(1, 86) = 5.96, *p* = 0.017 *	*F*(3, 46) = 2.24, *p* = 0.096	*F*(3, 46) = 1.44, *p* = 0.242	*F*(1, 68) = 13.88, *p* < 0.001 *
	Side > Anterior			
ROM at S1 (${}^{\circ }$)	*F*(1, 84) = 20.12, *p* < 0.001 *	*F*(3, 41) = 0.64, *p* = 0.595	*F*(3, 41) = 3.12, *p* = 0.036 *	*F*(1, 55) = 47.64, *p* < 0.001 *
	Side > Anterior		H, M, L: Side > Anterior	
Mean relative phase angle (${}^{\circ }$)	*F*(1, 122) = 0.07, *p* = 0.794	*F*(3, 60) = 5.23, *p* = 0.003 *	*F*(3, 59) = 0.40, *p* = 0.752	*F*(1, 86) = 2.17, *p* = 0.145
		NL > (L, M, H)		
**Sagittal plane**				
ROM at T6 (${}^{\circ }$)	*F*(1, 130) = 47.34, *p* < 0.001 *	*F*(3, 64) = 1.01, *p* = 0.393	*F*(3, 62) = 6.67, *p* = 0.001 *	*F*(1, 100) = 3.34, *p* = 0.071
	Side > Anterior		Side: (H, M) > NL	
			H, M, L: Side > Anterior	
ROM at S1 (${}^{\circ }$)	*F*(1, 120) = 0.287, *p* = 0.593	*F*(3, 58) = 0.12, *p* = 0.947	*F*(3, 57) = 0.36, *p* = 0.779	*F*(1, 112) = 0.79, *p* = 0.378
Mean relative phase angle (${}^{\circ }$)	*F*(1, 103) = 3.59, *p* = 0.061	*F*(3, 49) = 0.81, *p* = 0.493	*F*(3, 48) = 0.76, *p* = 0.523	*F*(1, 1) = 0.69, *p* = 1.0

**Table 5:** Experiment-2 (*n* = 10) estimated marginal mean ± SE for the No-load condition, and statistically significant mean differences ± SE relative to the no-load condition by carrying mode and load level, at average values of centered relative speed. Significant mean differences relative to the no-load condition by carrying mode across different load levels are within the square brackets (i.e., [- - mean ± SE - -]).

		Side Carry			Anterior Carry		
	No-Load	Low (4.5 kg)	Medium (9.1 kg)	High (13.6 kg)	Low (4.5 kg)	Medium (9.1 kg)	High (13.6 kg)
**Coronal plane**							
ROM at T6 (${}^{\circ }$)	4.3 ± 0.2	−0.4 ± 0.3	−1.1 ± 0.3	−1.0 ± 0.3	−0.4 ± 0.3	−1.1 ± 0.3	−1.0 ± 0.3
ROM at S1 (${}^{\circ }$)	8.3 ± 6.6	[ - - - - - - - - - - - - +0.3 ± 0.4 - - - - - - - - - - - - - - ]			[ - - - - - - - - - - - - −0.6 ± 0.4 - - - - - - - - - - - - - - ]		
Mean relative phase angle (${}^{\circ }$)	118.7 ± 6.2	+1.8 ± 7.1	+7.4 ± 7.1	+5.9 ± 7.1	−11.4 ± 7.7	−21.3 ± 8.0	−24.7 ± 7.8
**Transverse plane**							
ROM at T6 (${}^{\circ }$)	5.2 ± 0.3	[ - - - - - - - - - - - - +1.0 ± 0.3 - - - - - - - - - - - - - - ]			[ - - - - - - - - - - - - +0.2 ± 0.3 - - - - - - - - - - - - - - ]		
ROM at S1 (${}^{\circ }$)	7.1 ± 0.6	+1.3 ± 1.0	+2.5 ± 1.1	+2.3 ± 1.2	−0.7 ± 0.7	−0.9 ± 0.6	−0.9 ± 0.6
Mean relative phase angle (${}^{\circ }$)	66.1 ± 4.4	−18.1 ± 5.8	−20.7 ± 5.4	−17.8 ± 6.4	−18.1 ± 5.8	−20.7 ± 5.4	−17.8 ± 6.4
**Sagittal plane**							
ROM at T6 (${}^{\circ }$)	2.7 ± 0.1	+0.3 ± 0.2	+0.5 ± 0.2	+0.5 ± 0.2	−0.4 ± 0.2	−0.5 ± 0.2	−0.2 ± 0.1
ROM at S1 (${}^{\circ }$)	3.5 ± 0.3	-	-	-	-	-	-
Mean relative phase angle (${}^{\circ }$)	52.9 ± 3.3	-	-	-	-	-	-

**Figure A1:**
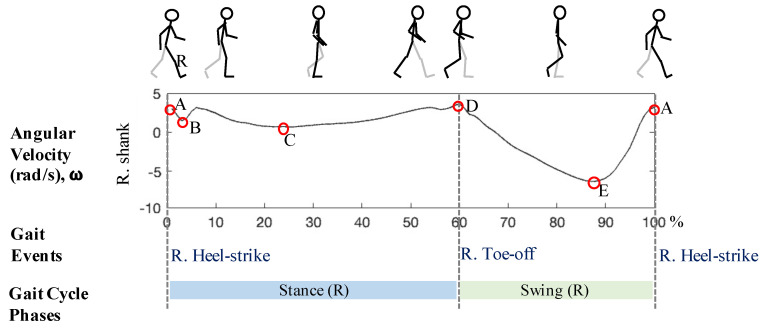
Graphical overview of the algorithm for detecting temporal gait events. Sample profile of the sagittal angular velocity (rad/s) from the right shank sensor is depicted for one complete gait cycle (100%) recorded during empty-handed condition. A = Right heel-strikes, B and C = minimum peaks within the right leg stance, D = Right toe-off, E = Mid-swing.

**Figure A2:**
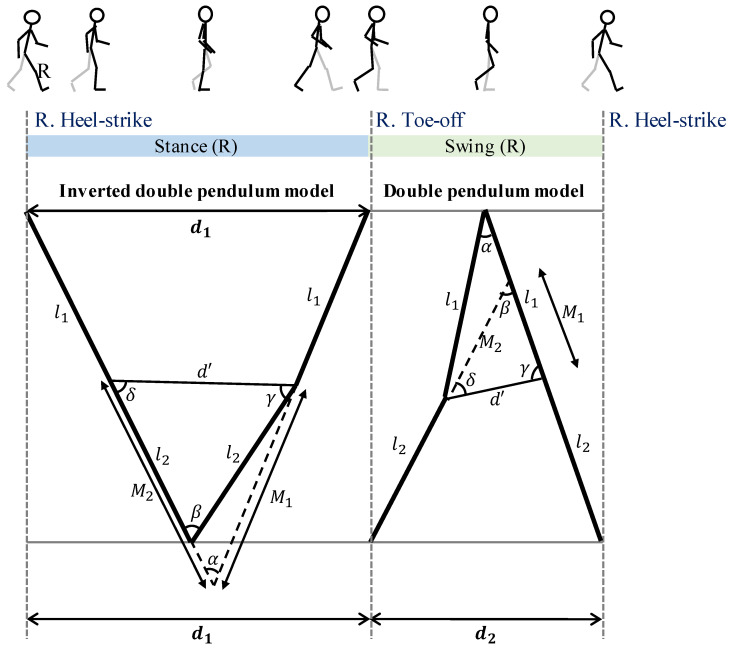
Graphical summary of the double pendulum model (adapted from Aminian et al. [42]) used for estimating the stride lengths from sagittal angular velocity (rad/s) data of the right shank and right thigh during right stance and swing phases separately.

**Figure A3:**
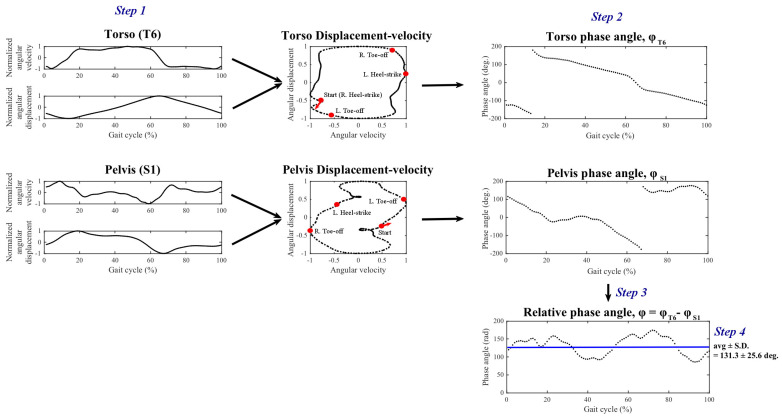
Overview of the algorithm for computing mean thoracic-pelvic relative phase angle using one exemplar participant data from the no-load condition in the coronal plane.

## Abbreviations

The following abbreviations are used in this manuscript:

IMU	Inertial Measurement Units
MMH	Manual Material Handling
MSD	Musculoskeletal Disorders
COM	Center of Mass

## Appendix A. Computing Temporal Gait Parameters Using Angular Velocity from a Shank-Mounted Gyroscope

Gait cycle duration was calculated as the time duration between right heel-strike to the consecutive right heel-strike (Figure A1). Detection of heel-strike and toe-off gait events were performed as the first step in a custom algorithm (modified from Aminian et al. [42]). One gait cycle was composed of the sequence of the following events: (1) right heel-strike, (2) left toe-off, (3) left heel-strike, (4) right toe-off, and (5) next right heel-strike. In this study, events (1), (4), and (5) were detected using sagittal angular velocity (rad/s) from inertial sensors on the right shank. The procedure is outlined below:

**Step 1: Detect mid-swing** 

Mid-swings were detected by finding the global minimum peaks (Points E in Figure A1) in the sagittal angular velocity (rad/s) data. To detect the valid global minima representing the mid-swing, Aminian et al. [42] applied a multi-resolution wavelet decomposition. In this paper, we applied two search criteria in terms of the amplitude of the peak and time duration between peaks. First, an amplitude threshold of −1.75 rad/s determined from preliminary testing was applied to identify the global minimum peaks of interest (i.e., Points E in Figure A1) while avoiding Points B after the heel-strike and the minimum peaks during the stance phase (i.e., Points C in Figure A1). Second, a time threshold of 0.63 s was used for the minimum time difference between consecutive global minima to ensure rejection of points B and C in Figure A1.

**Step 2: Estimating average gait cycle duration** 

Time duration between consecutive mid-swings were calculated to estimate the average gait cycle duration ($\bar{T}$^′^) using Equation (A1).

(A1) $${\bar{T}}^{\prime }=\frac{\sum_{0}^{N}\left(T{\left(E\right)}_{i+1}-T{\left(E\right)}_{i}\right)}{N}\;$$

where, *T*(*E*)${}_{i}$ is an array containing the timeframes for the *i*th instance of a mid-swing Point E, *N* is the number of gait cycles detected, and *i* = 0 to *N*.

**Step 3: Detect heel-strike** 

Heel-strikes were detected by finding the local maxima (Points A in Figure A1) within the range of [*T*(*E*)${}_{i}$ + 0.25 s, *T*(*E*)${}_{i}$ + $\bar{T}$^′^]. Multiple peaks may be detected within this time range so the search was refined in Step 5.

**Step 4: Detect Toe-off** 

Toe-offs were detected by finding the local maxima (Points D in Figure A1) within the range of [*T*(*E*)${}_{i}$ − $\bar{T}$^′^, *T*(*E*)${}_{i}$ − 0.05 s].

**Step 5: Refine heel-strike** 

Heel-strikes were refined amongst the detected heel-strikes from Step 3 by setting the search range of [*T*(*E*)${}_{i}$, *T*(*D*)${}_{i+1}$], where *T*(*D*)${}_{i+1}$ is the timeframe for i+1 instance of Point D. The first local peak after the mid-swing was selected as the heel-strike.

**Step 6: Pair heel-strike and toe-off events** 

Heel-strikes and toe-offs detected were paired if the time duration between heel-strike (*T(E)${}_{i}$*) to toe-off (*T*(*D*)${}_{i}$) was within [0.1 s, 2.5 s]. This pairing starts from the first heel-strike and toe-off in the time series and continued until the last heel-strike. Any heel-strike that does not meet this criterion was discarded.

**Step 7: Compute temporal duration between detected gait events** 

Time difference between consecutive heel-strikes (Figure A1, time duration combining each stance and swing in the bottom panel) were used to calculate gait cycle duration. Start and end of each stance and swing phase were used in estimating stride length in Appendix B.

## Appendix B. Estimating Stride Length from Angular Velocity of the Right Shank and Thigh Recorded by Gyroscopes

Stride lengths were estimated by algorithm adapted from Aminian et al. [42]. Sagittal angular velocity (rad/s) data from the right thigh ($\omega_{Thigh}$) and right shank ($\omega_{Shank}$) sensors and right thigh length ($l_{1}$, trochanter height − knee height) and right shank length ($l_{2}$, knee height) were used. The calculation was performed in a custom algorithm implemented in MATLAB R2016b (The MathWorks Inc., Natick, MA, USA). Leg movement during stance and swing phases were modeled as an inverted double pendulum and double pendulum [42], respectively, as depicted in Figure A2. During the right stance phase, the right shank and thigh rotate forward by pivoting the right foot on the ground. Although the foot has not moved forward, the body has moved forward by distance $d_{1}$. During the right swing phase the right foot steps forward by distance $d_{2}$. Total stride length can be estimated as the sum of $d_{1}$ and $d_{2}$ assuming bilateral symmetry, i.e., that right and left step lengths are equal. Stride lengths were calculated for each gait cycle *k* following the steps:

**Step 1: Calculate the distance** $d_{1}$ **moved during the stance phase**

The distance moved during the right stance phase ($d_{1}$) was estimated from the trigonometric relations. The angular rotation of the right thigh (α) and right shank (β) were estimated by integrating $\omega_{Thigh}$ and $\omega_{Shank}$, within each gait cycle, respectively. For each gait cycle *k*, we calculated $d_{1}\left(k\right)$ as follows:

(A2) $$d_{1}\left(k\right)=\sqrt{{\left(l_{1}+M_{2}\left(k\right)\right)}^{2}+{\left(l_{1}+M_{1}\left(k\right)\right)}^{2}-\left(l_{1}+M_{2}\left(k\right)\right)\left(l_{1}+M_{1}\left(k\right)\right)\cos\alpha \left(k\right)},\;$$

where:

(A3) $$\alpha \left(k\right)=\int_{R.Heel-strike(k)}^{R.Toe-off(k)}\omega_{Thigh}\left(t\right)dt,\;$$

(A4) $$\beta \left(k\right)=\int_{R.Heel-strike(k)}^{R.Toe-off(k)}\omega_{Shank}\left(t\right)dt,\;$$

(A5) $$M_{1}\left(k\right)=\frac{sin\delta (k)}{sin\alpha (k)}d^{\prime }\left(k\right),\;$$

(A6) $$M_{2}\left(k\right)=\frac{sin\gamma (k)}{sin\alpha (k)}d^{\prime }\left(k\right),\;$$

with:

(A7) $$\begin{array}{c} \gamma \left(k\right)=\frac{\pi -\alpha (k)}{2}\\ \delta \left(k\right)=\frac{\pi +\alpha (k)-2\beta (k)}{2}\\ d^{\prime }\left(k\right)=l_{2}\left(k\right)\sqrt{2(1-cos\beta (k))}\; \end{array}$$

**Step 2: Calculate the distance (**$d_{2}$**) moved during the swing phase** 

The distance moved during the right swing phase ($d_{2}$) was estimated as follows:

(A8) $$d_{2}\left(k\right)=\sqrt{{\left(l_{2}+M_{1}\left(k\right)\right)}^{2}+{\left(l_{2}+M_{2}\left(k\right)\right)}^{2}-\left(l_{2}+M_{1}\left(k\right)\right)\left(l_{2}+M_{2}\left(k\right)\right)\cos\beta \left(k\right)},\;$$

where:

(A9) $$\alpha \left(k\right)=\int_{R.Toe-off(k)}^{R.Heel-strike(k)}\omega_{Thigh}\left(t\right)dt,\;$$

(A10) $$\beta \left(k\right)=\int_{R.Toe-off(k)}^{R.Heel-strike(k)}\omega_{Shank}\left(t\right)dt,\;$$

(A11) $$M_{1}\left(k\right)=\frac{sin\delta (k)}{sin\beta (k)}d^{\prime }\left(k\right),\;$$

(A12) $$M_{2}\left(k\right)=\frac{sin\gamma (k)}{sin\beta (k)}d^{\prime }\left(k\right),\;$$

with:

(A13) $$\begin{array}{c} \gamma \left(k\right)=\frac{\pi -\beta (k)}{2}\\ \delta \left(k\right)=\frac{\pi -2\alpha (k)+\beta (k)}{2}\\ d^{\prime }\left(k\right)=l_{2}\left(k\right)\sqrt{2(1-cos\alpha (k))}\; \end{array}$$

**Step 3: Combining** $d_{1}$ **and** $d_{2}$

Stride length is the total distance moved during one gait cycle *k*:

(A14) $$Stride\;length\left(k\right)=d_{1}\left(k\right)+d_{2}\left(k\right)\;$$

## Appendix C. Algorithm for Calculating the Mean Thoracic-Pelvic Relative Phase Angle

Mean relative phase angle [34] was calculated using the angular velocity (rad/s) data obtained from the torso ($\omega_{T6}$) and pelvis ($\omega_{S1}$) sensors. Additionally, angular displacement (rad, θ) was calculated by integrating the angular velocity data across time within each gait cycle. The algorithm is outlined below along with an example obtained during no-load condition depicted in Figure A3.

**Step 1: Calculate normalized angular velocity and angular displacement** 

Normalized angular velocity and angular displacement were obtained by rescaling the amplitudes of the data to the range [−1, 1] within each normalized (%) gait cycle separately for each sensor.

**Step 2: Calculating phase angle** 

Phase angles of the torso and pelvis segments (ϕ) were calculated separately by following Equation (A15):

(A15) ϕ(t)=arctan(ω(t)/θ(t))

where ω(*t*) represents the normalized angular velocity time series, θ(*t*) the normalized angular displacement, at *t* = 0, ..., 100%.

**Step 3: Calculate relative phase angle** 

Relative phase angle between torso and pelvis was obtained following Equation (A16):

(A16) $$\Delta \phi \left(t\right)=\phi_{T6}\left(t\right)-\phi_{S1}\left(t\right)\;$$

**Step 4: Calculate mean relative phase angle** 

Mean relative phase angle, which was the dependent measure used in the present study, was computed as the average of the relative phase angle over one gait cycle:

(A17) $$\Delta \bar{\phi }=\frac{\sum_{t=0}^{N}\Delta \phi \left(t\right)}{N}\;$$
